# Eye and head movements in visual search in the extended field of view

**DOI:** 10.1038/s41598-024-59657-5

**Published:** 2024-04-17

**Authors:** Niklas Stein, Tamara Watson, Markus Lappe, Maren Westendorf, Szonya Durant

**Affiliations:** 1 Institute for Psychology, University of Münster, 48143 Münster, Germany; 2 Otto Creutzfeldt Center for Cognitive and Behavioral Neuroscience, 48143 Münster, Germany; 3 MARCS Institute for Brain, Behaviour and Development, Western Sydney University, Sydney, NSW 2751 Australia; 4 Department of Psychology, Royal Holloway, University of London, Egham, TW20 0EX UK

**Keywords:** Visual search, Virtual reality, Head movements, Eye movements, Search strategy, Pattern matching, Human behaviour, Psychology

## Abstract

In natural environments, head movements are required to search for objects outside the field of view (FoV). Here we investigate the power of a salient target in an extended visual search array to facilitate faster detection once this item comes into the FoV by a head movement. We conducted two virtual reality experiments using spatially clustered sets of stimuli to observe target detection and head and eye movements during visual search. Participants completed search tasks with three conditions: (1) target in the initial FoV, (2) head movement needed to bring the target into the FoV, (3) same as condition 2 but the periphery was initially hidden and appeared after the head movement had brought the location of the target set into the FoV. We measured search time until participants found a more salient (O) or less salient (T) target among distractors (L). On average O’s were found faster than T’s. Gaze analysis showed that saliency facilitation occurred due to the target guiding the search only if it was within the initial FoV. When targets required a head movement to enter the FoV, participants followed the same search strategy as in trials without a visible target in the periphery. Moreover, faster search times for salient targets were only caused by the time required to find the target once the target set was reached. This suggests that the effect of stimulus saliency differs between visual search on fixed displays and when we are actively searching through an extended visual field.

## Introduction

Detailed knowledge of how we search an array of visual stimuli for a unique target has mostly been acquired using stimuli that onset in front of a participant on a computer monitor. Participants are often instructed to keep their heads still and initially focus their eyes on a central point on the screen. Outside of controlled experimental conditions like these, we often search for items beyond our current field of view (FoV). The work presented here compares visual search when targets are presented within the visual field at the start of a search, to when they appear due to the act of moving the head and eyes to bring them into view. We compare the effect of salience on visual search under these circumstances, anticipating that a more salient stimulus entering the FoV after a head or eye movement would assist the participant in orienting to this stimulus.

Experiments limiting search to a fixed FoV have provided a detailed understanding of how target stimuli are located amongst distractor stimuli. Some targets are found with very little delay after onset within the visual field, regardless of the number of distractors also presented. These are said to ‘pop out’ relative to surrounding distractor objects, which means they are found without the need to make any exploratory saccadic eye movements, or within only a single saccade^1^. Targets that pop out are differentiated from distractors by some basic attribute (e. g. opposing colours)^2–4^. Otherwise, an effortful search must take place. This involves serially selecting/inspecting each stimulus or ‘clumps’ of stimuli^5^. This kind of ‘serial search’ is suggested to occur by establishing a priority map^6,7^, which is a planned sequence of locations to allocate attention toward and thereby explore ordered by the weighted average of the bottom-up salience, top-down feature guidance, scene guidance, historical performance, and value according to past reward^4^. Serial search can be undertaken by shifting covert attention (i.e. by making no overt gaze change) via a priority map of stimuli that can be ascertained within the current functional FoV^8^. The functional FoV can be considered to encompass the region of the visual field that is resolved well enough that stimuli can be identified for response or rejection without making a gaze shift and is therefore a smaller region than the entire FoV. Outside of the functional FoV, the priority map may also take into account what we know about the likely location of any particular target object within that scene. In this case, previous knowledge in the form of spatial memory becomes an input to the search plan. This is referred to as contextual cueing^9^.

Visual search involving a search array that extends beyond the FoV of the participant, and therefore requiring head and eye movements, has been less often investigated. To achieve an extensive search array like this, studies must be carried out beyond a 2D computer monitor. Stimuli need to be situated in the real world or presented via a simulation; either through immersive simulators or virtual reality (VR). In this study we use VR to present a search array extending beyond the participant’s initial FoV. Previous studies have investigated visual search in a VR environment and found effects transferred from 2D screens in terms of the overall effect on reaction times of target-distractor discriminability^10,11^. Shioiri et al. have shown that contextual cueing builds up across trials even when the search display covers $360^{\circ }$ and the participant must look around themselves to find the targets^12^. As yet, the question of whether more salient targets are able to support a pop-out-like search when the target comes into view due to the participant’s own movement during an ongoing search has not yet been systematically investigated. This is despite the fact that it is of great importance for guiding the attention of users in virtual and natural environments.

On the one hand, Lukashova-Sanz and Wahl showed that blurring salient regions of a visual scene can guide the observer’s attention away from those regions and enable better search performance^13^. On the other hand, studies using naturalistic search stimuli or tasks suggest eye movements are not guided by salience as predicted via image statistics^14–16^. In addition, contextual cues and memory of the likely location of targets have been shown to play an important role in search strategy in naturalistic virtual environments^17,18^. This opens up the possibility that stimuli found to ‘pop-out’ when appearing in the FoV at the start of a visual search trial may not ‘pop-out’ when they appear in peripheral vision due to a head turn or eye movement. However, naturalistic environments present a complex salience landscape, whereas traditional visual search experiments tightly control the appearance of the visual scene. As such, pop-out may not be changed in this context, despite extensive FoV shifts.

In the following experiments we investigate visual search beyond the initial FoV by asking participants to find a target stimulus presented within one of four search ‘sets’ presented in VR. The ‘sets’ are arranged such that two were presented in the periphery at the start of the trial (inner panels) but the remaining two are only viewable after participants make a large eye or a head movement (outer panels). Therefore, this is a controlled environment within which participants are instructed to freely move their head and eyes to find more or less salient targets as quickly as possible among distractors that are placed around them.

**Figure 1:**
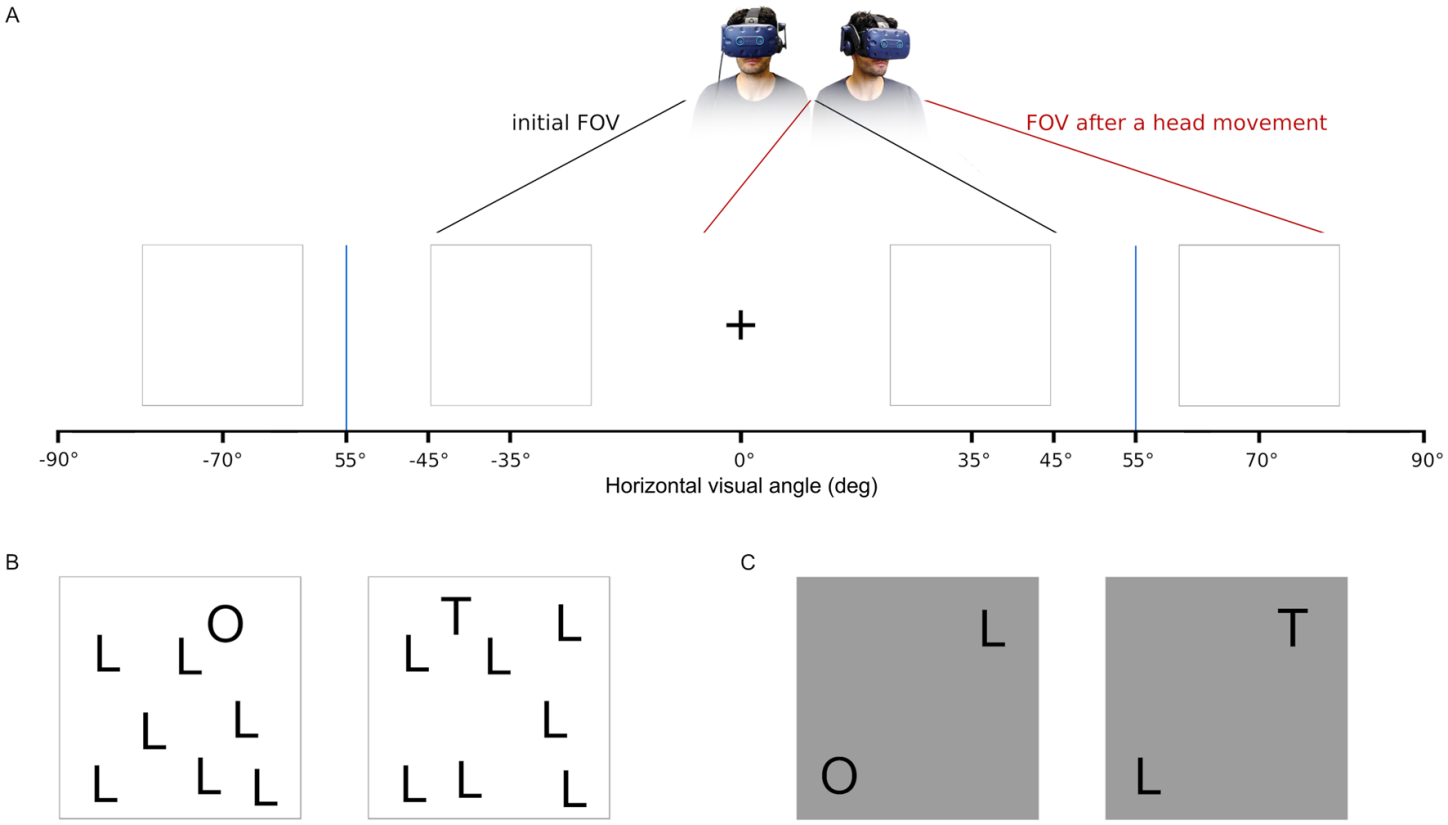
(**A**) Schematic illustration of the experiments. Participants were instructed to find a target, either a ‘more salient’ letter O or ‘less salient’ letter T amongst distractors (L) that were arranged in four sets, two of which were visible in the initial FoV while the other two were outside of it. There was only one target in each trial. In the hidden set condition in Experiment 2, the letters in outer locations appeared after gaze passed the corresponding invisible threshold at $55^{\circ }$ (blue line). (**B**) In Experiment 1, each set contained 8 letters. Sets were presented in a uniform grey environment, the bounding box around each set ($19.6^{\circ } \times 19.6^{\circ }$) was not visible and 2D images were projected on a sphere using equirectangular projection. (**C**) In Experiment 2, each set was presented as a gray 2D panel ($19.6^{\circ } \times 19.6^{\circ }$) with visible borders containing two letters spaced $4.9^{\circ }$ and $-\,4.9^{\circ }$ from the center of the panel. The sets were positioned in a uniform gray environment facing the participant.

We report two experiments that differ in the number of distractor stimuli presented on each panel. The first experiment shows eight stimuli per set while the second experiment presents a reduced set of two stimuli (see Fig. 1). Additionally, in Experiment 2, we introduce a condition in which the stimuli on the outer panels are hidden until the participant’s gaze is directed almost directly at the panel. Both experiments aim to answer what the effect of salience of a peripheral stimulus on saccade and head movement planning during visual search in an extended FoV is.

## Experiment 1

### Methods

#### Participants

35 participants (18–25 years old, $\hbox {M} = 20.1$, $\hbox {SD} = 1.3$, 17 female, 18 male) were recruited via a combination of personal approach, and online advertising to gain course credit/or a £5 reimbursement. 5 participants were excluded according to data quality criteria (see below in “Data analysis” section), leaving 30, enough for 80% power and a medium effect size ($dz = 0.4$) for a 2 $\times$ 2 repeated measures ANOVA (27 participants required)^19^. All had normal or corrected to normal vision and gave written informed consent to the participation on the study. The study adhered to the tenets of the Helsinki Declaration (2013). Experimental procedures were approved by the Royal Holloway, University of London Ethics Committee.

#### Equipment

Experiment 1 was conducted in the VR lab of the Psychology Department of Royal Holloway, University of London. Stimuli were presented on an HTC Vive headset retrofitted with Tobii eye trackers with a resolution of $1080 \times 1200$ pixels per eye, a frame rate of 90 Hz and a nominal FoV of $110^{\circ }$, which was connected to a Lenovo ThinkStation P510 desktop with an NVIDIA Quadro M5000 graphics card. The virtual display was created by drawing 2D $3840 \times 1920$ pixel images which were presented by Tobii Pro Lab ver. 1.130 software as a $360^{\circ }$ image using equirectangular projection in the HTC Vive headset display. Two Vive Lighthouses 1.0 were used to track the head movements and the position of the headset in the room. Head and eye movements were recorded using Tobii Pro Lab software, gaze output frequency was 120 Hz. During typical usage we expected eye tracking accuracy of $1.08^{\circ }{-}2.74^{\circ }$ for the Vive Pro Eye^20^.

#### Procedure and stimuli

All participants were seated on a standard office swivel chair that could be rotated $360^{\circ }$ and the headset was fitted. They were told they could swivel the chair round during the experiment. The cabling was laid out in such a way that the participant could move their heads freely and without resistance. Participants were given a practise session in which four trials were shown, one each O/T, inner and outer.

At the start of the experiment, after a 5 point eye tracker calibration, four crosses were shown, arranged in a square around the fixation cross location and the participant was asked to look at each in turn (in no particular order). This was partially to make sure the participant was facing in the correct direction, so that the fixation cross would appear in front of them and was also used as a calibration check.

The virtual search environment was arranged in a fixed layout that was consistent throughout the whole experiment (see Fig. 1). A fixation cross was presented at $0^{\circ }$ of horizontal and vertical angle straight ahead. At $35^{\circ }$ (inner) and $70^{\circ }$ (outer) left and right from the fixation cross were sets of letters, each containing 8 letters randomly spaced out within a defined area. Inner sets were visible peripherally from the start, outer sets could only be reached through a head movement. In each trial participants had to start at the fixation cross and search as fast as possible for a more salient (O) or less salient (T) target in an environment with 31 distractors (L). They were asked to fixate it and press any button on the keypad in their hand. The keypad was not visually represented in the virtual environment. Participants were not aware whether the target would be an ‘O’ or ‘T’ on each trial. After the button response the stimuli for that trial disappeared and only the central fixation cross remained on screen in the intervals between each trial for 3 s. At the end of the experiment the four crosses appeared again with the same instruction as at the start. To further ensure eye tracking data quality, the experimenter remained present and monitored how the stimuli appeared to the participant, their real-time gaze location and the Tobii Pro Lab visual indicator of how well the eyes were being tracked.

Within one experimental run the target appeared in each of the sets 10 times, yielding 2 (O/T) $\times$ 4 (set location) $\times$ 10 = 80 trials. This resulted in 20 measurements per condition (O/T and inner/outer). Five different full experimental stimuli were pre-generated (randomizing the locations of the letters within sets each time) and each participant saw one of these. The order of stimuli was randomly shuffled for each participant.

#### Data analysis

To stabilise an object on the retina e.g., when the head catches up with an eye movement after a saccade, the eyes then move into the opposite direction of the head^21,22^. Therefore, in contrast to head fixed experiments a fixation can not be identified by just using the eye tracking data and a standard velocity threshold. Instead, the sum of eye and head movement (gaze) needs to be analyzed.

The raw gaze data was filtered using the default Tobii Pro Lab I-VT attention filter with 60 ms minimum fixation duration and the velocity threshold parameter set to $100^{\circ }/\hbox {s}$. The reported time of a fixation was the first time point at which a gaze fixation was identified. Gaze contingent stimulus presentation was not possible, so trials were excluded post-hoc if the fixation at the start of the trial was further than $9.4^{\circ }$ (100 pixels) away from the fixation cross. This arbitrary threshold was chosen based on inspection of trials containing obvious non-compliance with task instructions. Each fixation was assigned a ‘set’ label according to the set the nearest letter belonged to (having moved more than $9.4^{\circ }$ from fixation). We use this label to report the time of the start and the distance from target of the first fixation within a set and the last fixation within a set. If none of the final three fixations before responding were in the set containing the target, or the participant never fixated within $9.4^{\circ }$ of any letter, these trials were also excluded (as incorrect trials). Participants with more than 10% of trials excluded were fully excluded.

### Results

Altogether from the 30 participants included, 3.6% of the trials were excluded, and we used 2313 trials for further analysis. On average over all trials there was a median search time (to final fixation on target set) of 1.36 s (1. Qu 0.83 3. Qu 2.22) and it took a median time of 2.09 s (1. Qu 1.50 3. Qu 2.87) until the button response. Median button response search times averaged across participants for the inner target locations were 1.48 s (more salient targets) and 2.11 (less salient targets). For the outer target locations search times until button response were 2.52 s (more salient targets) and 2.77 s (less salient targets).

Based on final fixation time on the target set, targets in inner sets were found faster (median = 0.84 s) than targets in outer sets (median = 1.97 s) and more salient targets (median = 1.10 s) were found quicker than less salient targets (median = 1.84 s).

#### First search direction

**Figure 2:**
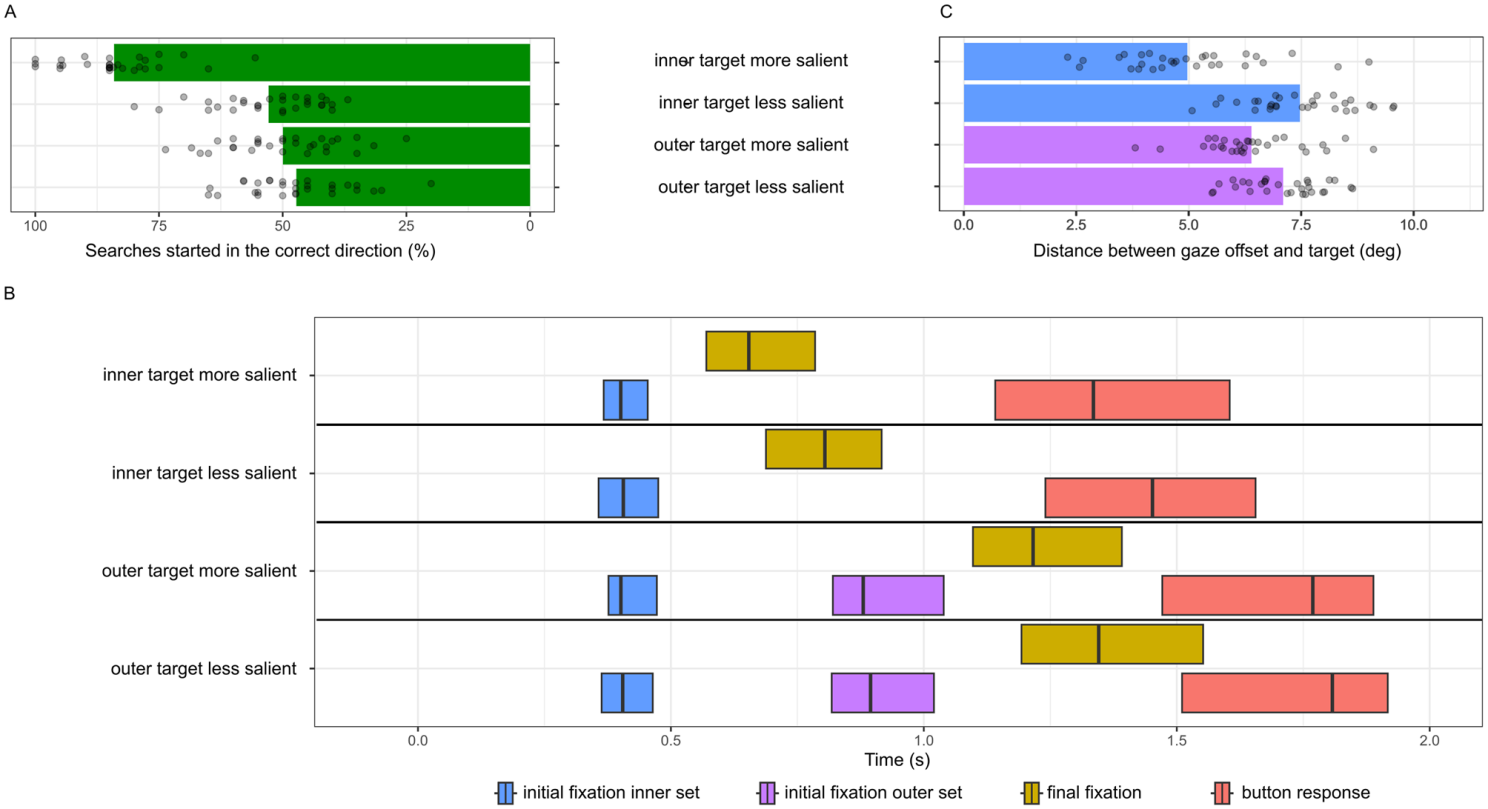
(**A**) Percentage of trials in which participants started the search in the target direction based on the position of the first fixations in Experiment 1. (**B**) Averaged timing of key events in searches across all participants in Experiment 1. Vertical lines show median times, box thresholds indicate lower and upper quartiles. (**C**) Distance between the location of the initial fixations on the target set and the target in Experiment 1 (for all trials).

As a first step we analyzed whether the direction of the first gaze movement went in the correct direction in all searches (Fig. 2A). Only when a salient target was in an inner set were participants able to reliably initiate their first gaze direction change in the correct direction: When the target was in an inner set, a paired t-test revealed a significant difference between the more salient (*M* = 84.1%) and less salient (*M* = 52.9%) condition ($t = 12.78, df = 29, p < 0.0001$), while the confidence interval of the less salient condition overlapped chance level (50%). As expected, in searches with outer targets (that were not initially in the FoV) the proportion of correct first gaze movements was at chance level and there was no significant difference between the two salience conditions (*M* = 50% and *M* = 47.2%) ($t = 0.94, df = 29, p = 0.35$).

#### Timing of head-, eye- and gaze movements

To be able to summarize search times from different trials in a sensible way, we sorted all trials based on the order in which participants looked at the different sets. Participants reliably searched in one of the inner sets first (98.0% of trials). If the target was not in the inner set, they then typically moved their gaze to the outer set on the same side (72.6%) (based on the location of the first gaze movement on a set). In 90% of trials the target was found after 4 sets were inspected. In 73% of trials the target was found after 3 sets were inspected. In 61% of trials the target was found after two sets were inspected. In 34% of trials the target was found after 1 set was inspected.

We defined two common types of searches that we analysed further. The first category were search trials in which participants started their search in the correct direction and found the target in the first set they looked at (minimal search with target in the inner location). The second category included trials in which participants started their search in the correct direction, did not find a target at the inner set and continued their search in the same direction to find a target there (minimal search with target in the outer location). In these trials we then defined the timing of the first saccade offset (i.e. fixation onset) on the inner set, the start of final fixation in the set containing the target, and the button response. For the minimal outer searches, we also defined the timing of the saccade offset on the outer set (i.e beginning of first fixation on the outer set). The three gaze related timings were defined based on the fixation filter from the Tobii software (see Experiment 1 Methods—“Data analysis” section).

Figure 2B shows the results of the gaze timing analysis for minimal searches. Since search times are naturally skewed, we used non-parametric tests to compare the individual means of the two salience conditions.

For the first category, participants initiated a gaze change with the same reaction time no matter the stimulus but took less time to complete their search to a salient target. A paired Wilcoxon rank sum test for the initial fixation time on the inner set showed no significant difference between the more salient ($\hbox {median} = 0.4\,s$) and less salient ($\hbox {median} = 0.41\,\hbox {s}$) condition ($W = 450, p = 0.99$). There was a significant difference between final fixations on the target set in trials with more salient ($\hbox {median} = 0.65\,\hbox {s}$) than less salient ($\hbox {median} = 0.8\,\hbox {s}$) targets in the inner set ($W = 240, p = 0.00161$).

For the minimal searches with an outer target, there was no effect of salience on search timing. A paired Wilcoxon rank sum test indicated no significant difference between the salient ($\hbox {median} = 0.88\,\hbox {s}$) and less salient ($\hbox {median} = 0.89\,\hbox {s}$) condition ($W = 444, p = 0.93$) for the initial fixation time on the outer set. There was also no significant difference between final fixations in trials with more salient ($\hbox {median} = 1.22\,\hbox {s}$) and less salient ($\hbox {median} = 1.35\,\hbox {s}$) targets ($W = 352, p = 0.15$).

#### Gaze landing position

As a final step we analyzed distances of the landing points of the gaze movements from the target of all trials from the experiment (Fig. 2C). Participants were able to use the more salient stimulus to better guide gaze behaviour toward the target in both the inner and outer sets. When the target was in an inner set, a paired t-test revealed a significant difference between the more salient ($M = 5^{\circ }$) and less salient ($M = 7.5^{\circ }$) condition ($t = 7.64, df = 29, p < 0.0001$). In minimal searches with outer targets a paired t-test also indicated a small significant difference between the more salient ($M = 6.4^{\circ }$) and less salient ($M = 7.1^{\circ }$) condition ($t = 3.37, df = 29, p = 0.002127$).

### Discussion

We find that for the inner targets the results are as expected. In terms of search times, the more salient targets are detected from the fixation cross more often and are found more quickly. Analysis of landing positions and timing of the final fixation from minimal searches (where gaze moved in the correct initial direction) confirms that the gaze lands more accurately on target for more salient inner targets.

For outer targets however, although in the minimal searches (where gaze continues in the correct direction to outer targets) the gaze landing position for the more salient targets is still a little more accurate, a more salient target does not significantly decrease the time it takes to move the eyes towards the target set. Although it seems clear that peripheral information could influence gaze planning, we found no evidence for this in search time.

We considered whether our results might be due to the difficulty of finding either type of target, perhaps not allowing for the effects of salience to be fully seen. The lack of ability to extract real time fixation measures and provide feedback led to participants not always fixating accurately on target, meaning we had to use a measure of ‘final fixation on target set’ rather than ‘final fixation on target’, which is a less accurate measure of gaze based search time. These considerations led to Experiment 2.

## Experiment 2

In Experiment 2, we reduced the number of distractors per set to make the peripheral target even more visible, potentially enhancing the effect of saliency. We also wanted to find out whether the effects of salience weakened when stimuli were not visible in the periphery after the first change in eye position. Therefore, we added a new *hidden* condition in which outer sets became visible after gaze crossed $55^{\circ }$ on the corresponding side.

### Methods

#### Participants

Thirty-three participants took part in the study (18–35 years old, M = 22.3, SD = 3.6, 23 female, 10 male). Participants received student credits or 4€ for taking part in the experiment. All participants had normal or corrected-to-normal vision. They gave written informed consent to the participation on the study. The study adhered to the tenets of the Helsinki Declaration (2013). Experimental procedures were approved by the Ethics Committee of the Department of Psychology and Sports Science of the University of Münster.

#### Equipment

The experiment was conducted at the Department of Psychology and Sport Science at the University of Münster. Stimuli were presented on an HTC Vive Pro Eye headset with a resolution of $1440 \times 1600$ pixels per eye, a frame rate of 90 Hz and a nominal FoV of $110^{\circ }$, which was connected to an MSI GE63VR 7RF Raider laptop with an NVIDIA GTX 1070 graphics card. The virtual environment was created using Unity3D and the Unity Experiment Framework UXF^23^. Two Vive Lighthouses 2.0 and the gyroscope of the HTC Vive Pro Eye were used to track the head movements and the position of the headset in the room. During the entire experiment head and eye tracking data was collected with a target frequency of 90 Hz.

#### Procedure and stimuli

All participants were instructed to face towards a desk with a keyboard on it and sit in a steady straight position at the beginning of each trial. They sat on a chair that could be rotated $360^{\circ }$ and whose height they were allowed to adjust. The cabling was laid out in such a way that the participants could move their heads freely and without resistance. Experiment 2 was a re-implementation of the previous experiment as an interactive 3D environment instead of a $360^{\circ }$ image. Moreover, the custom software enabled more control over the head and eye tracking data to produce further insight into the search behaviour. At the start of the experiment participants followed the eye tracking default calibration procedure implemented in the SRAnipal Software (version 1.3.6.8) of the head mounted display.

Again, participants were asked to search for a more salient (O) or less salient (T) target as fast as possible. The environment contained a total of 7 distractors (L) evenly distributed across 4 panels. Participants were asked to fixate the target and press the space bar of an external keyboard in front of them. The keyboard was not visually represented in the virtual environment, therefore participants were instructed to keep one hand on the space bar during the whole experiment. The visual environment was arranged similarly to the first experiment (Fig. 1A). At the beginning of a search a fixation cross was presented at $0^{\circ }$ of horizontal angle and participants were asked to fixate it while keeping the head steady and facing forward. At $35^{\circ }$ (inner) and $70^{\circ }$ (outer) left and right from the fixation cross were two grey panels each. After the fixation cross was fixated for 0.5 s the trial started and the fixation cross disappeared. Now, each panel showed two letters in fixed positions (see Fig. 1C). Again, the inner sets were visible peripherally from the start, the outer sets could only be seen after a head movement. In the *hidden set* condition objects in the outer locations only became visible after their gaze passed a threshold of $55^{\circ }$ on the correspondent side.

Each participant performed 320 trials in which an ‘O’ or a ‘T’ was presented at one of the 8 possible positions. Each type of trial was repeated 10 times. Target positions were balanced and presented in a pseudo-random order. Trials in which the participant did not gaze at the correct target when pressing the space bar were counted as error trials and were repeated at the end of the session. Errors could also result from participants not fixating the correct target or inaccurate eye tracking of a fixation of the correct target. As before, we expected an accuracy of $1.08^{\circ }{-}2.74^{\circ }$ for the Vive Pro Eye during typical use^20^, which was expected to be accurate enough to fixate the targets used in this study. However, because potential headset slippage could have resulted in invalid data, the eye tracker was automatically recalibrated every time 10 error trials were reached before the next trial was started.

#### Data analysis

For Experiment 2 we used the raw eye movement and head movement data to define gaze movements and use the offset of these on the letter sets for the search timings. To get a valid gaze and gaze velocity signal, eye- and head tracking data need to be perfectly synchronized to each other. Otherwise, eye and head movements in opposite directions would result in a noisy gaze signal and overly large velocities during gaze fixations. Based on previous studies^24^ and pilot studies of participants fixating a target while moving the head using the HTC Vive Pro Eye, we assumed that the eye tracking data measured with Vive Pro Eye headsets is delivered with some delay. Therefore, we shifted the eye tracking data 4 frames ($\sim 44\,\hbox {ms}$) back and calculated gaze as the sum of the head- and shifted eye tracking data.

### Results

Data was collected at an average frame rate of 89.3 Hz. Overall, participants had to repeat 14.76% error trials, because they did not fixate the correct target when pressing the space bar. These trials were excluded from further analysis, resulting in 320 trials per participant. In 9.53% of trials, participants moved their head faster than $15^{\circ }/\hbox {s}$ at the start of the trial (− 100 ms to the start of the search). During the same period, in 5.49% of the trials, the head was not aligned with the center (more than $10^{\circ }$ away from $0^{\circ }$). In 9.83% of the trials participants did not fixate the fixation cross stably enough at the start of the trial. 3.11% of trials included missing eye tracking data for more than 100 ms at the start of the trial. Therefore we excluded 22.085% of trials and used 8227 search trials for the further analysis.

On average participants needed a median search time (to final fixation on target) of 0.94 s (1. Qu 0.53 s 3. Qu 1.76 s) and took a median time of 1.56 s (1. Qu 1.14 s 3. Qu 2.31 s) until the button response. Median search times averaged across participants for the inner more salient targets were 0.48 s (1. Qu 0.37 s 3. Qu 0.62 s) and 0.6 (1. Qu 0.46 s 3. Qu 0.83 s) for less salient targets. For the outer target locations the median search time was 1.63 s (1. Qu 1.0 s 3. Qu 1.65 s) for more salient targets and 1.66 s (1. Qu 1.12 s 3. Qu 2.13 s) for less salient targets. In the hidden condition at outer target locations the median search time was 1.75 s (1. Qu 1.12 s 3. Qu .21 s) for more salient targets and 1.67 s (1. Qu 1.16 s 3. Qu 2.18 s) for less salient targets.

Typically, the search was initiated with a synchronous head and eye movement. However, in some rare cases, participants made one saccade towards one of the inner sets at the start of the search while keeping the head still and then continued the search with a combined head- and eye movement to the opposite side.

Participants reliably searched on one of the inner sets first (91%). If the target was not in the inner set, they then typically moved their gaze to the outer set on the same side (61%). In 92% of trials the target was found after 4 sets were inspected. In 76% of trials the target was found after 3 sets were inspected. In 64% of trials the target was found after two sets were inspected. In 37% of trials the target was found after 1 set was inspected.

**Figure 3:**
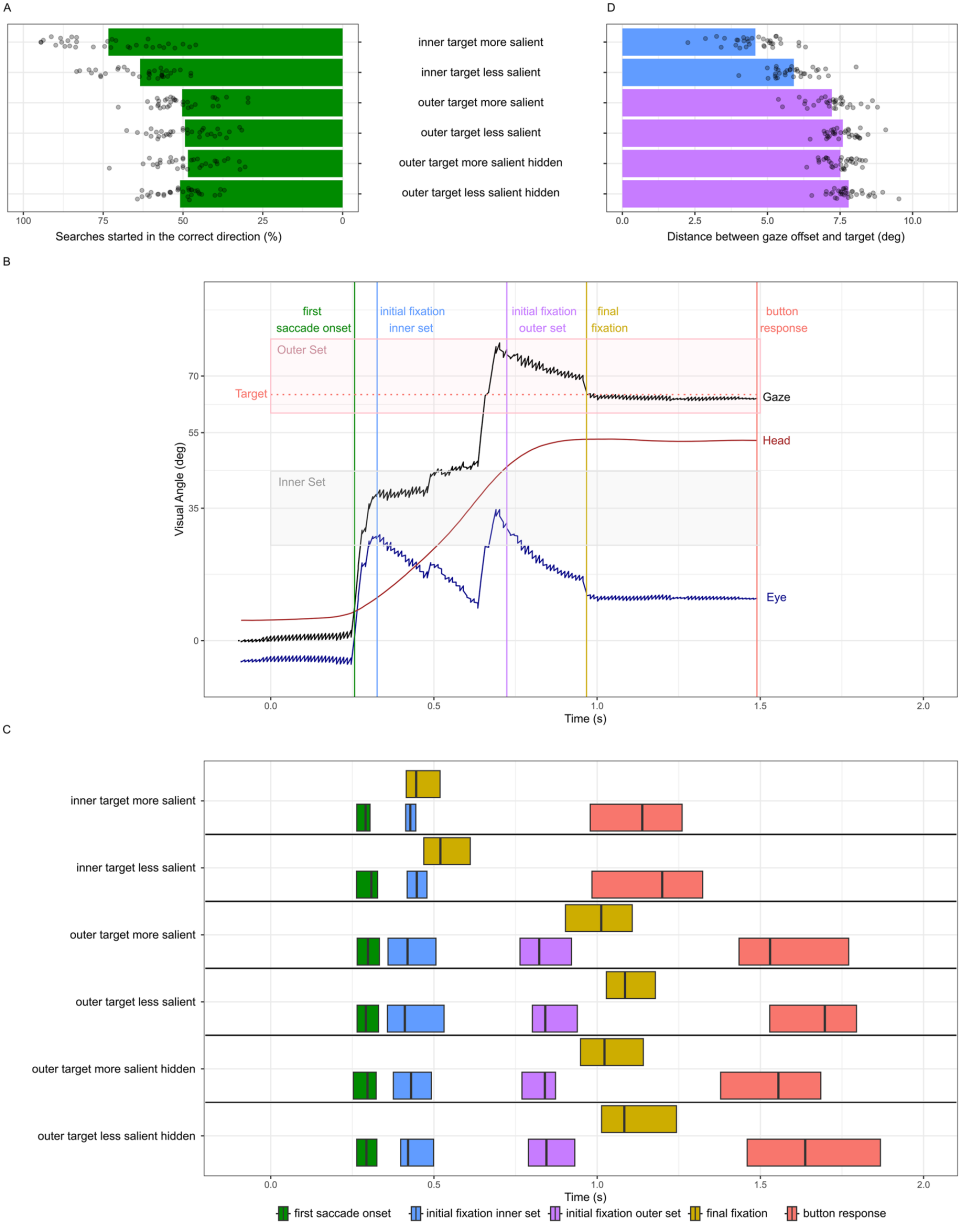
(**A**) Percentage of trials in which participants started the search in the target direction in Experiment 2. (**B**) Sample trial of gaze-, head and eye movements during a minimal search for a salient target in an outer set. Key properties are represented as vertical lines. (**C**) Averaged timing of key events in minimal searches across all participants in Experiment 2. Vertical lines show median times, box thresholds indicate lower and upper quartiles. (**D**) Distance between initial fixations on the target set and the target position in Experiment 2 (for all trials).

#### First search direction

As a first step we analyzed whether the direction of the first gaze movement went in the correct direction in all searches. First search direction was defined based on a $10^{\circ }$ threshold around the fixation cross. When the target was in an inner set, a paired t-test revealed a significant difference between initially moving gaze in the correct direction in the more salient ($M = 73.3\%$) and less salient ($M = 63.6\%$) condition ($t = 3.07, df = 56.362, p = 0.003$), while neither confidence interval overlapped chance level (50 %). In searches with outer targets the proportion of correct first gaze movements was at chance level for all conditions as expected (means (confidence interval from one sample t-test, $df = 32$): more salient = 50.1% (46.8%, 53.3%), less salient = 49.5% (46.1%, 52.9%), more salient hidden = 48.5% (45%, 51.9%), less salient hidden = 50.9% (48.3%, 53.5%), all $p > 0.05$) and therefore there was no difference between any of the conditions. Fig. 2 A shows an overview.

#### Timing of head-, eye- and gaze movements

Again, we defined two common types of searches that we analysed further. In these, participants either inspected an inner set and found a target, or inspected an inner and outer set on the same side and found a target at the outer location (see Fig. 3B for a sample trial).

Differentiating clearly between fixations, saccades and eyes that move opposite to an ongoing head movement in free headed experiments is challenging^25^. To detect fixations, every eye tracking sample was labeled based on collisions of gaze ray and objects in the virtual environment. We then calculated a gaze velocity signal and smoothed it with a Gaussian filter with a sigma of 5 samples. Fixations were then defined based on a velocity threshold of $50^{\circ }/\hbox {s}$. Only gaze sequences with the same label for more than three samples were kept as eye movement offsets/fixations.

Across all searches from the first and second category, the first gaze movement was detected at 0.3 s. The following fixation on the inner cluster was $33.4^{\circ }$ away from the center and was detected at on average 0.442 s. In minimal searches with an outer target the participants’ gaze then remained on the first inner set for a mean time of 0.656 s at an average horizontal position of $36.4^{\circ }$. Then participants typically initiated a big gaze movement to reach the outer panel.

To compare trials from both categories, we defined the following key properties in each trial (see Fig. 3B): (1) *First saccade onset* the first sample in which the standard deviation of the horizontal eye angle on a moving window of 100 ms starting from trial start reached a threshold of $1^{\circ }$. (2) *Initial fixation on inner panel* the first sample labeled as fixation in which gaze was on one of the inner target sets. (3) *Initial fixation on outer panel* the first sample labeled as fixation in which gaze was on one of the outer target sets. (4) *Final fixation* the first sample of the final fixation on the target object, before the button response was given. (5) *Button response* the first sample with a button response while fixating the target.

Since search times are naturally skewed, we used non-parametric tests to compare these key properties. Participants were able to use the salience of the target, as seen from the periphery, to undertake a faster search. For the initial fixations on the inner set there was a significant difference between the more salient (median = 0.43 s) and less salient (median = 0.44 s) condition ($W = 416, p = 0.014$). For inner searches a paired Wilcoxon rank sum test revealed also a significant difference between the timing of final fixations in trials with more salient (median = 0.45 s) and less salient (median = 0.52 s) targets in the inner set ($W = 532, p < 0.001$).

Participants were not able to speed up the time it took to undertake gaze changes to bring a salient peripheral target into the direct FoV. For the initial fixations on the outer set with outer targets a Friedman test for paired rank sum group comparisons indicated no significant difference ($\chi ^2 = 3.58, p = 0.31$, $df = 3$) between any of the conditions (medians: more salient = 0.82 s, less salient = 0.84, more salient hidden = 0.84 s, less salient hidden = 0.84 s).

Once gaze was directed at the set containing a target, the more salient target did reduce search time. For the final fixation in searches with outer targets a Friedman test for paired rank sum group comparisons indicated significant differences ($\chi ^2 = 24.75, p < 0.001, df = 3$) between the conditions (medians: more salient = 1.01 s, less salient = 1.09 s, more salient hidden = 1.02, less salient hidden = 1.08). Bonferroni corrected posthoc paired pairwise Wilcoxon rank sum tests revealed significant differences between more salient vs less salient ($p < 0.001$).

For this to occur, the target did not need to be visible in the periphery prior to directing gaze toward the target containing set. Bonferroni corrected posthoc paired pairwise Wilcoxon rank sum tests revealed significant differences between more salient hidden vs less salient hidden ($p = 0.011$), less salient vs more salient hidden ($p = 0.019$) and more salient vs less salient hidden ($p < 0.001$).

Participants performed the same whether or not they had the opportunity to ’see’ the target in periphery while gazing at the inner set (see Fig. 3C). There were no significant differences between more salient vs more salient hidden ($p = 0.16$) and less salient vs less salient hidden ($p = 0.99$).

#### Gaze landing position

As a final step we analyzed distance of the landing points of the initial fixation from the target of all trials from the experiment. When the target was in an inner set, a paired t-test revealed a significant difference between the more salient ($4.6^{\circ }$) and less salient ($5.9^{\circ }$) condition ($t = 8.44, df = 32, p < 0.0001$). In searches with outer targets a paired t-test also indicated a small difference between more salient ($7.2^{\circ }$) and less salient ($7.6^{\circ }$) condition($t = 3.03, df = 32, p = 0.005$). When outer targets were hidden, there was no significant difference between more salient and less salient targets ($t = 1.93, df = 32, p = 0.06202$). Figure 3D shows an overview.

Therefore, the peripherally visible, more salient target, when it appears under traditional search conditions, was better able to guide accurate fixations within the set. Like in Experiment 1, we also found a small increase of accuracy when the target was in an outer set. As expected, salience did not influence the gaze landing point within the outer set in the hidden set condition.

### Discussion

The results are similar to Experiment 1. With a reduced number of stimuli, gaze moves initially in the correct direction a significant proportion of times even when there is a less salient target in an inner set in this case, but still an even higher proportion of times for salient targets. The gaze landing point within the set is also closer to the target for salient targets. Thus, participants were able to use the available peripheral information of the target to guide their initial search behaviour. Participants’ gaze change behaviour also indicated that they were able to identify the more salient target faster when it is in an inner set than less salient targets. This is expected from typical visual search findings on 2D screens.

A timing difference between more salient and less salient targets was also observed for targets in the outer sets. This was the case whether or not the target was available to enter the FoV from the start of the trial or required gaze to be beyond $55^{\circ }$ before the outer set’s onset (*hidden set*). Despite an overall search time advantage for more salient targets, the gaze behaviour across both conditions showing targets in the outer sets confirms this advantage mostly emerges after the participants are directly inspecting the set containing the target. This means that salience doesn’t contribute to a faster search time at the time the target set appears in the periphery during a search.

Like in Experiment 1, participants initial gaze did land closer to more salient targets in outer sets. However, when comparing the landing distance between the inner and outer sets, the overall accuracy was still worse for the outer sets, even though the number of stimuli was reduced compared to the previous experiment. This suggests again that peripheral information might have been available before an outer set was approached. To investigate whether this information was used by the participants for their search strategies, we set up an ideal observer model.

## Ideal observer model

To analyse the participants’ search strategy, we set up an ideal observer model that derives decisions based on two latent parameters. The first, $\gamma$, describes the proportion of times that a participant saccades in the direction of a target in the periphery when there is no information from the periphery to guide their decision. Thus, $\gamma$ could be considered a guess rate but it could also model a bias, if participants tend to prefer one direction over another, for example continuing on the same side for the second decision as for the first. Specifically, $\gamma$ may vary across different target locations, due to the layout of the stimulus. The second parameter, $\sigma$, describes the proportion of time that the participant makes a saccade to a peripheral target when the target is present, over and above what could be expected based on $\gamma$. Thus, $\sigma$ is a measure of the behavioral effects of the target, i.e., how often the target guides the decision. Salient targets should result in high $\sigma$ values, as they make it more likely that a target is seen in the periphery. Less salient targets should lead to a smaller $\sigma$-value. The combination of $\sigma$ and $\gamma$ determines the percentage of correct decisions $P_c$:

1 $$\begin{aligned} P_c = \gamma + (100-\gamma ) \times \frac{\sigma }{100}. \end{aligned}$$

The two parameters can be estimated from the data of the first decision, i.e., when the participant sees the two inner panels, which may or may not contain a target. If the panels do not contain a target, $\sigma$ is 0 and choice is driven by $\gamma$. Based on the setup of the stimuli we expect $\gamma$ at the start of the search to be $\approx$ 50% as the two locations to chose from are equidistant and the target is equally likely to occur either side. Indeed, in both experiments, when there was no target in the inner locations the proportion of correct first gaze movements was at chance (Experiment 1: $\gamma = 48.5\%$ (46.0%, 51.1%); Experiment 2: $\gamma = 49.7\%$ (48.9%, 50.6%), for single conditions see Experiment 2: First Direction and Fig. 2A). Experiment 2: First Direction and Fig. 3A).

Looking at the percentage correct ($P_c$) data from trials that did contain a target at an inner location we can then calculate $\sigma$ for the two salience conditions (more salient and less salient) by inverting Eq. (1):

2 $$\begin{aligned} \sigma = \frac{P_c -\gamma }{100-\gamma } \times 100. \end{aligned}$$

In Experiment 1, participants initially went to a location in the correct direction in $P_c = 52.9\%$ (48.8%, 56.9%) of trials when a less salient target was present in one of the inner locations. This equates to a value of $\sigma = 5.5\%$ (− 4.9%, 15.9%). In trials with a more salient target in inner locations participants chose the correct location in $P_c = 84.1\%$ of trials ($\sigma = 67.9\%$ (59.8%, 75.9%)). Therefore, they were able to use the peripheral information at the start of the trial when a more salient target was present.

In Experiment 2, participants initially went in the correct direction in $P_c = 63.6\%$ (60%, 67.2%) of trials when a less salient target was present, which results in $\sigma = 30.6\%$ (23.7%, 37.6%). When a more salient target was present participants initially went in the correct direction in $P_c = 73.3\%$ (67.9%,78.7%) of trials, which results in $\sigma = 49.1\%$ (38.8%, 59.4%). Therefore, they were (as expected) able to use the peripheral information at the start of the trial in both salience conditions.

**Figure 4:**
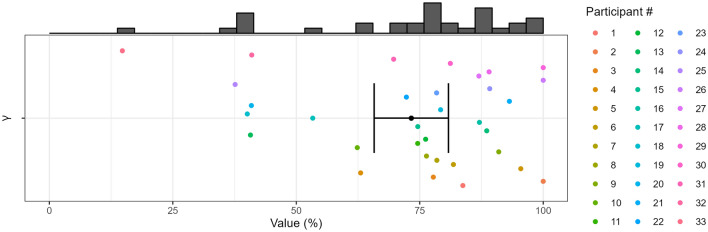
Distribution of $\gamma$ of the second decision for all participants of Experiment 2. Individual values of $\gamma$ were estimated using the results from the hidden condition. They represent how often a participant continued their search on the same side as in the first decision when no information about targets in the outer set was available. The black dot represents the mean across participants, the black error bar represents the confidence interval.

We can now use the ideal observer model to consider the next step of a search, when the target is not present in the inner locations. We consider what should happen after gaze has moved to one of the inner locations in the correct direction, i.e. the target is now present in the peripheral outer location, e.g., the target is in a set at $74.9^{\circ }$ and the observer has moved their gaze to $35^{\circ }$. This, hence, is the second decision to be taken by the participant. Here the value of $\gamma$ is less clear a priori, as the layout is less balanced. For example, the observer might have a bias to carry on towards the outer location rather than changing direction. This would be reflected in a biased value of $\gamma$ for the second decision. We can estimate $\gamma$ for the second decision from the data in the hidden condition in Experiment 2, in which no information about the outer target was present before the saccade to the outer panel was initiated. In this condition, participants on average continued their search in the same direction (i.e. with a saccade to the outer panel) in $\gamma = 73.3\%$ (69.7%, 76.9%) of trials (Fig. 4). Hence, our participants were strongly biased towards continuing their search in the same direction as in the first decision.

**Figure 5:**
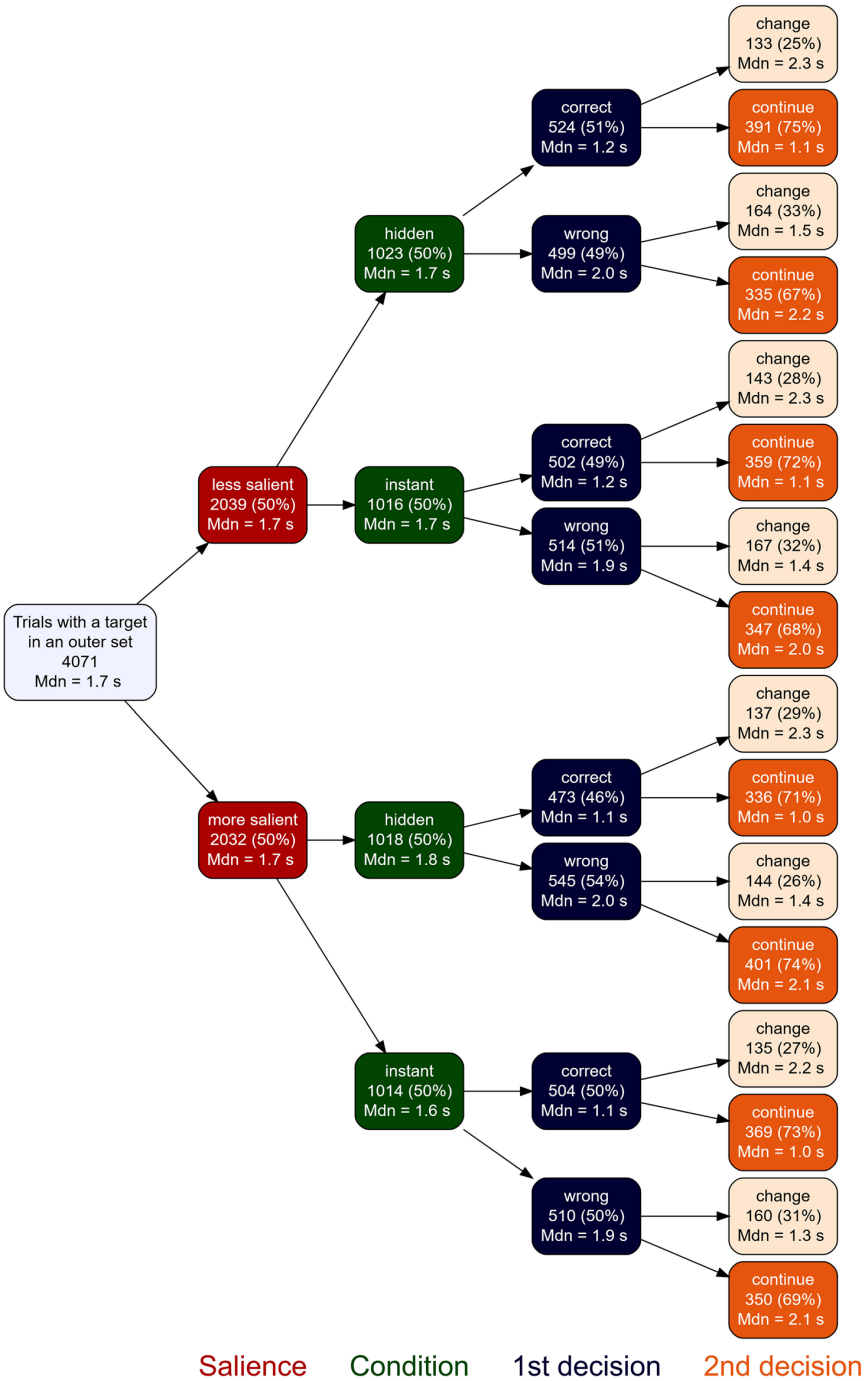
Trials with a target on an outer set including median search times (Mdn) and the absolute and relative amount of trials in each condition and decision. While the first decision (blue) was made at random, on the second decision (orange) participants preferred to continue their search on the same side. However, in 25–33% of trials with a target in an outer set, they switched sides after having previously inspected only an inner set.

How much should target salience then affect choice probability in the second decision? If salience is used in the same way as in the first decision at the start, we can use the values for $\sigma$ estimated from the first decision to calculate an expected percentage correct for the second decision from Eq. (1). Using the previous $\sigma$ values of each participant to predict $P_c$, the model predicts an average value of 84.7% (80.4%, 89%) in the more salient condition and 80.6% (76.1%, 85%) in the less salient condition. These predictions are clearly at odds with the data: the measured $P_c$ in Experiment 2 remain very similar to the 73.3% mark both for more salient (73.9% (68.3%, 79.4%)) and less salient (71.8% (66.5%, 77%)) targets present in the periphery. Thus, our observers did not follow an ideal observer strategy, or they could not use saliency in the second decision in the same way as in the first decision (for an overview of all decisions and all search times in all conditions see Fig. 5).

Experiment 1 did not include a hidden condition and we cannot compute $\gamma$ for the second decision in the same way as in Experiment 2. However, we may use data from trials in which no target was present on the corresponding side (because the first decision was wrong) to estimate $\gamma$ for the second decision and then predict $P_c$ based on the $\sigma$-values obtained from the first decision. When no target was present in the outer location, participants continued on the same side in 71.2% (62.6%, 79.8%) of trials. This is close to the $\gamma$ calculated from the hidden condition in Experiment 2 (73.3%). Then, from the $\sigma$ estimated in the first decision on the more salient target (67.9%) the model predicts $P_c = 89.8\% (83.3\%, 96.2\%)$ for the second decision with the more salient target. As in Experiment 2 these predictions are not consistent with the observed data ($P_c= 77.7\%$ (69.1%, 86.3%)). For the less salient target, $\sigma$ estimated in the first decision was already very small (5.5%), indicating little effect of salience already at the start of the search in this experiment. Consequently, the model prediction for $P_c$ was determined mostly by $\gamma$. The model predicted $P_c = 73.4\%$ (64.1%, 82.7%), which is both in the range of $\gamma$ (71.2%) and in the range of the observed data ($P_c = 77.8\%$ (68.6%, 87%)). In summary, therefore, the values for $\sigma$ obtained from the first decisions in either experiment provided poor predictions for $P_c$ of the second decision, indicating little effect of salience when the target was in an outer set.

## General discussion

We tested whether there was a difference in visual search for stimuli that became visible in the periphery by appearing at the start of a trial versus those locations that only became visible in the periphery as the participants moved their eyes and heads to inspect the scene. We designed two experiments in which participants were required to choose between and inspect up to four sets of letters across different locations, in order to detect a target. These sets were arranged such that two were visible to participants when the trial began, whereas two required participants to move their head to bring them into view. We manipulated the salience of the targets, the number of distractors, and how the stimuli become visible to investigate the way in which the location and salience of peripheral targets influences the planning of search strategy and the associated landing positions. Analysis of gaze dynamics highlights key differences in the effect of salience on visual search when targets were in the initial FoV versus when they appear in the FoV due to head and eye movements.

When targets appeared in the FoV at the start of the trial, both experiments showed faster identification of the more salient target. Participants were requested (Experiment 1) or required (Experiment 2) to fixate the target when responding, which involved making a large change of gaze direction to complete the task. Accordingly, both experiments showed participants reliably made an initial gaze movement in the direction of the salient target. When there were two stimuli per set participants still reliably made an initial gaze direction change in the direction of the less salient target. With 8 stimuli per set, participants were unable to reliably make their initial gaze in the correct direction of the less salient target. The ability to better identify the more salient target from the periphery was accompanied by a gaze movement that landed closer to the target.

We anticipated that a stimulus capable of supporting ‘pop out’-like search, as found when the target was in the inner sets, would also facilitate faster target detection when the stimulus appeared in the FoV on the basis of the participant’s own head and eye movements as part of the ongoing search task. We expected participants would first fixate an inner set and from there a more salient target would allow participants to accurately and immediately continue on to the outer target set, facilitating faster target fixation in these trials. This was not the case. Both experiments showed that it took equally long for participant’s gaze to arrive at the correct outer set no matter the target saliency, even when we reduced the stimuli to two per set. Experiment 2 also introduced a *hidden set* condition whereby the outer sets only appeared after gaze direction crossed $55^{\circ }$ of visual angle, to further test if peripheral information had any influence on gaze movements. This was not the case. There was no difference in search performance whether the outer sets were available in the environment throughout the whole trial or appeared under the hidden set condition, suggesting that the process to reach the target cluster differed between inner and outer sets.

In addition, we found that participants’ gaze lands closer to the target on salient trials than on less salient trials (Experiment $1 = -2.5^{\circ }$, Experiment $2 = -1.3^{\circ }$). However, this difference becomes smaller for the outer target sets (Experiment $1 = -0.7^{\circ }$, Experiment $2 = -0.4^{\circ }$), while the absolute distance between initial gaze landing point and target is also higher in these sets. This also indicates that the participants approach sets that are brought into the FoV during an ongoing search differently.

The more refined gaze direction analysis in Experiment 2 showed that once participants had fixated within the correct outer set, more salient stimuli were responded to faster, which is in line with previous studies on visual search without head movements in a fixed FoV. Thus, our results show a difference between visual search when stimuli appear in the periphery as in a traditional visual search experiment, relative to when they are brought into the FoV by a head movement as part of the ongoing search task. In other words, a highly salient stimulus shown to ‘pop out’ from the periphery under the same conditions as typical visual search, does not show this property when it enters the FoV due to the participants’ own head and eye movements as part of an ongoing search task. The fact that there is no difference between the two outer target conditions in Experiment 2 (hidden vs always visible), further confirms that peripheral information was not used in this experiment during the search task after the initial gaze change has occurred.

There are two possible explanations for participants apparently not using the peripheral information about the stimulus to reach the target faster after they have started to actively look around the stimulus sets. The first is that they are unable to use the peripheral visual information in the same way as they could at the start of the trial. The second is that participants were able to gather the presence of the more salient stimulus from the periphery but did not use this information to guide their search behaviour.

Participants may have been unable to see the peripheral stimuli when they entered the FoV because they were actively shifting their gaze at this time. For example, it could be that the gaze on the inner set was never stable enough or at the equivalent distance from the target-containing set relative to fixation. We find when inspecting the more detailed gaze tracking data in Experiment 2, that participants do gaze on an inner set for sufficient time and at sufficient proximity to the outer set. Figure 3B exemplifies the typical gaze on the inner set prior to moving to the outer set containing the target. It also shows a pattern we observed that suggests that despite gazing at the inner set for a period, the participant had decided to inspect the outer set at the point of making the first gaze change. The example trial shows one large head movement with the eyes acting to stabilise the gaze on the two sets around the single large head movement. This behaviour was common across participants and indicates participants may have decided on a course of action already after the initial view of the stimuli.

Although saliency alone has been shown not to predict gaze direction when freely viewing $360^{\circ }$ environments in VR^26^, in our experiments the salient target was relevant to the task and the visual scene was controlled as in traditional visual search experiments. Therefore, we expected the reduced visual search environment and the task relevance of the salient stimulus would allow salience to guide search. This did not occur consistently across stimulus locations, suggesting that salience even when relevant to the task is not the key predictor of gaze direction when active exploration is occurring around a scene with strong spatial layout cues. In studies with more natural environments, other factors like memory^18^ and scene context^27,28^ have been shown to influence searching for objects in a simulated real world context in virtual reality. Other research has also suggested that encoding of stimulus salience and its utility in driving gaze behaviour in a naturalistic scene is time limited and diminishes throughout a trial^29–31^. In addition, it has been shown that saccades with a short latency are guided by salience, while saccades with a long latency are more likely to take value information into account^32^. Visual search in the extended FoV could therefore be influenced by the use of strategies. However, these search patterns could also make use of salience.

In order to find out whether target salience affects search strategy, we then analyzed two key decisions during each search trial and compared them to an ideal observer model: first, the initial search direction and, second, the decision whether to keep on searching on the initially taken side vs changing sides. The ideal observer model was based on two parameters: $\gamma$, an indicator of uninformed choice bias, and $\sigma$ an indicator of choice based on target salience. The results indicated that $\sigma$ influenced the first decision as expected, providing a measure of the salience of each target. However, $\sigma$ had little predictive power for the second decision, indicating that the second decision was not affected by salience. Instead, the second decision was dominated by $\gamma$, a bias to continue search on the same side in 73% of trials, no matter whether a salient target appeared in the periphery or not.

This indicates that the participants followed a predetermined strategy in the second decision, rather than using peripheral vision to guide their search. If no peripheral information is gathered for the second condition an ideal observer should decide between continuing on the same side and changing sides based on minimal effort and time loss. We can estimate effort based on the median search times of Experiment 2. The search time advantage of continuing the search on the same side, vs changing sides after inspecting the first set was 0.18 s. Searches with an outer target in which the search was continued at the second decision took a median search time of 1.61 s, searches with an outer target in which the search direction was changed at the second decision took a median search time of 1.79 s. Shifting gaze from an inner panel to an outer panel ($35^{\circ }$) took on average 0.17 s while shifting gaze from one inner panel to the other inner panel ($70^{\circ }$) took on average 0.22 s. Hence, continuing on the same side leads to a slightly faster search on average. Therefore, an ideal observer should always follow this strategy when no peripheral information is used. However, our participants continued search on the same only 73% of the time. This is not ideal behaviour. Yet, similar patterns, following non-ideal strategies in two and threefold decisions before have been described in humans^33,34^ and animals^35,36^ before. While an ideal observer would always minimize the effort, distance and trial time and should therefore choose the ideal move in every trial, humans often deviate from this behaviour and follow a strategy that can be described as pattern matching. In pattern matching participants show a discrete behavioral pattern that asymptotes at the probability of success instead of always picking the most probable choice. The occurrence of pattern matching is quite common and has shown to be influenced by the effort needed to implement a strategy^37^, reward (increased task reward makes pattern matching less likely)^38^, and task expectations (for example setting a local focus to look at each trial separately makes the occurrence of pattern matching less likely)^39^. Cognitive load during the decision does not change the likelihood that participants use pattern matching^40,41^. After a large number of trials, participants typically overshoot the initially observed probability of success^42^. Therefore, pattern matching can also be described as part of a learning trajectory that could at some point lead to the ideal strategy of maximizing the ideal choice and therefore going for the best option in every trial^43^.

Looking at the distribution of $\gamma$ in Fig. 4, we can see that although three participants reached an individual $\gamma$-value of 100% (and therefore always continued on the same side at the second decision) most participants show individual $\gamma$-values around 75%. In our experiments in particular, once the participant has determined that the target is not in any of the inner sets, the distance to the set on the opposite side is three times larger than the distance to the set on the same side ($105^{\circ }$ vs $35^{\circ }$). Therefore, a strategy that matches behavior to the expected upcoming gaze movement distance could result in 75% staying on the same side vs 25% switching sides.

Overall, our results indicate that saliency plays a subordinate role in visual search in the extended FoV. In our two experiments, participants selected where to look based on information from the periphery only at the beginning of the search. All subsequent directional decisions appeared to be selected largely depending on the known arrangement of the stimuli. It is possible that the structure of the environment, alongside stored memory content, knowledge about the current task, the expected reward and cues from the environment are the more important information for the ongoing active exploration of our environment. This could also explain why salient objects are not always found at first sight when searching for them in the real world. However, this does not mean that salience plays no role at all. Once gaze has been directed to a set by head movements, the familiar mechanisms known from classic visual search paradigms take effect and salient stimuli simplify the last part of the search.

The use of VR technology has enabled us to bring experimental control to a less constrained FoV, however we cannot say for certain that viewing behaviour in this virtual space is not changed by the constraints imposed by the smaller FoV or the weight of the headset, for example. We also observed interesting differences between Experiment 1 that did not use a gaze contingent design and Experiment 2 that measured whether participants were looking at the target when they responded via the keyboard. Experiment 1 presented a challenge for data analysis as we observed participants were often eager to return their gaze to the fixation cross before responding but were likely unaware of this. More detailed gaze data analysis in Experiment 2 was facilitated by the gaze contingency, however this controlled behaviour is a departure from the more naturalistic behaviour found in Experiment 1. While we feel these differences do not interfere with the findings in this research, it is important to keep in mind that fully virtual worlds introduce the necessity to explicitly make the trade-off between producing data structured via experimental control such that complex tasks can be aligned for analysis and allowing the participant to interact naturally with the environment. Our final observation is the importance of awareness of the temporal shift between the different sensors available in commercial headsets. The constraints on each sensor used to track a person in virtual space are that they produce a good enough experience for the user. Our experiences show that this may not be good enough for research purposes and temporal alignment cannot be taken at face value. With these caveats in mind, we feel there is great value in taking well established visual and cognitive tasks off 2D screens and enacting them in virtual three-dimensional or immersive situations.

In summary, using VR to measure visual search in an extended FoV we found that salient targets do not produce ‘pop-out like’ search when they enter the FoV after participants have started to move their gaze around the scene. This suggests that visual search using a traditional visual search array does not occur in the same way when active exploration outside the initial FoV is necessary. Instead participants follow a pre-planned gaze sequence likely informed by knowledge of the layout of the scene.

## Data Availability

All data is available in an open science framework repository https://osf.io/ewsj5/.
